# Submacular Hemorrhages Show No Significant Seasonal Variations in a European Cohort

**DOI:** 10.3390/jcm12113622

**Published:** 2023-05-23

**Authors:** Jens Julian Storp, Raphael Diener, Nicole Eter, Eike Bormann, Maximilian Treder

**Affiliations:** 1 Department of Ophthalmology, University of Muenster Medical Center, 48149 Muenster, Germany; 2 Institute of Biostatistics and Clinical Research, University of Muenster, 48149 Muenster, Germany

**Keywords:** anticoagulation, AMD, bleeding, blood pressure, retina, seasons, size

## Abstract

The aim of the article is to investigate the seasonality of acute submacular hemorrhages (SMHs) in a European population and analyze the influence of the seasons, arterial hypertension, and intake of anticoagulatory/antiplatelet (AC/AP) medication on hemorrhage size. This retrospective, monocentric study included 164 eyes of 164 patients treated for acute SMH at the University Hospital Münster, Germany, between 1 January 2016 and 31 December 2021. Data on the day of occurrence, hemorrhage size, and general patient characteristics were recorded. “Test for cyclic trends in incidence data” and the Chi-Square Test were applied to investigate seasonal variations in SMH incidence. Fisher’s exact test was used to investigate the influence of the seasons, arterial hypertension, and intake of AC/AP medication on hemorrhage size. A statistical analysis did not reveal significant seasonal variations in the occurrence of SMHs (*p* = 0.81). While the seasons and the presence of systemic arterial hypertension did not exert a significant influence, the intake of AC/AP medication significantly affected the size of SMH (*p* = 0.03). In this European cohort, no significant seasonal variations of SMHs were observed. However, in patients with risk factors, such as neovascular age-related macular degeneration (nAMD), the chance of an increase in hemorrhage size should be considered when initiating AC/AP therapy.

## 1. Introduction

Submacular hemorrhages (SMH) represent a rare but potentially sight-threatening complication of various retinal diseases, such as neovascular age-related macular degeneration (nAMD) [1]. Despite different surgical approaches, visual outcome is poor in many cases [2,3]. Untreated, SMH typically leads to a permanent and severe loss of vision ranging from 6/30 to light perception [4]. Therefore, various studies have investigated incidence and risk factors of occurrence as well as outcomes after SMH to optimize treatment regiments or even prevent their occurrence [5].

Although novel imaging modalities, such as optical coherence tomography (OCT), have substantially broadened our understanding of SMH, some aspects, such as seasonal fluctuations in the occurrence of hemorrhages, remain unclear. A great number of vascular diseases are associated with certain risk factors, which in turn are known to show seasonal variations [6,7,8,9,10]. This aspect raises the question of possible seasonal variations in the occurrence of SMH. In 2006, a Japanese publication demonstrated significant seasonal variations in the occurrence of acute SMH associated with AMD and found a winter-dominant prevalence [11]. However, in a more recent study conducted by the same research group, these results could not be reproduced. The authors attribute this finding to the enhancements in ophthalmological diagnostics and treatment of the last decade, which has seen the development of OCT devices and anti-vascular endothelial growth factor (VEGF) drugs [12]. There is currently a lack of further trials investigating this issue. Furthermore, current evidence is only derived from Asian populations. It is unclear whether the results currently available would also apply to other groups consisting of different ethnicities. The contradictory nature of trial findings as well as the limitation of trials to Asian populations have made the investigation of seasonal variations in the occurrence of SMH in other regions necessary.

Thus, the primary aim of this trial is to investigate a possible seasonal effect in the incidence of SMHs in a European cohort. The secondary objective is to report the influence of the season of occurrence, the presence of arterial hypertension as a concomitant disease, and the intake of anticoagulatory (AC) and antiplatelet (AP) medication on the size of SMH.

## 2. Materials and Methods

### 2.1. Design and Setting

This was a monocentric, retrospective study consisting of data from patients presenting with acute SMH at the Department of Ophthalmology at Münster University Hospital—A tertiary eye care center—Between 1 January 2016 and 31 December 2021. The study was approved by the ethics committee of the Medical Association of Westfalen-Lippe and the Westphalian Wilhelms University Münster (project number: 2022-487-f-S) and complies with the principles of the Declaration of Helsinki. Due to the retrospective nature of the study, the local ethics committee deemed the obtainment of informed consent not necessary.

### 2.2. Data Sources and Measurements

Retrospective evaluation of patient data was performed using the integrated search function within the digital documentation systems FIDUS (Arztservice Wente GmbH, Darmstadt, Germany) and ORBIS (ORBIS SE, Saarbruecken, Germany). Patient files containing the terms “macular hemorrhage” and “macular bleeding” as well as the International Statistical Classification of Diseases (ICD-10) codes H35.3 and H35.6 and the operation and procedure codes (OPS) 5–154.3, 5–156.9 and 5–158 were compiled and reviewed.

Information on the time of onset, localization, and underlying disease of eyes with SMH, as well as the cardiovascular status, intake of AC and AP medication, age, gender, and history of systemic hypertension from the patient that was affected were extracted from the electronic patient records. Patients with incomplete files or missing fundus photography were not eligible for inclusion. Eyes with previous macular hemorrhage, hemorrhages that did not originate from the macula, or which occurred due to diabetic retinopathy, were excluded as well.

### 2.3. Primary and Secondary Objectives

The primary objective was to assess the seasonal variations in the occurrence of SMHs. We defined December, January, and February as winter; March, April, and May as spring; June, July, and August as summer; and September, October, and November as autumn. The secondary objective was to analyze the influence of the season of occurrence, arterial hypertension as a concomitant disease, and intake of AC/AP medication on the size of SMH.

The size of the hemorrhage was determined by two expert examiners (JJS and RD) on the basis of fundus photographs on the first day of presentation (VISUCAM, Zeiss, Germany, and CLARUS 500, Zeiss, Germany) and was based on the consensus of the Vision Academy on the Management of Subfoveal Hemorrhage [13]:

▪ Small: hemorrhage size equals 1–≤4 disc diameters
▪ Medium: hemorrhage size equals >4 disc diameters, not extending beyond the vascular arcades
▪ Large: hemorrhage extends beyond the temporal vascular arcades, but not past the equator
▪ Massive: hemorrhage extends past the equator in at least two quadrants

**Figure 1:**
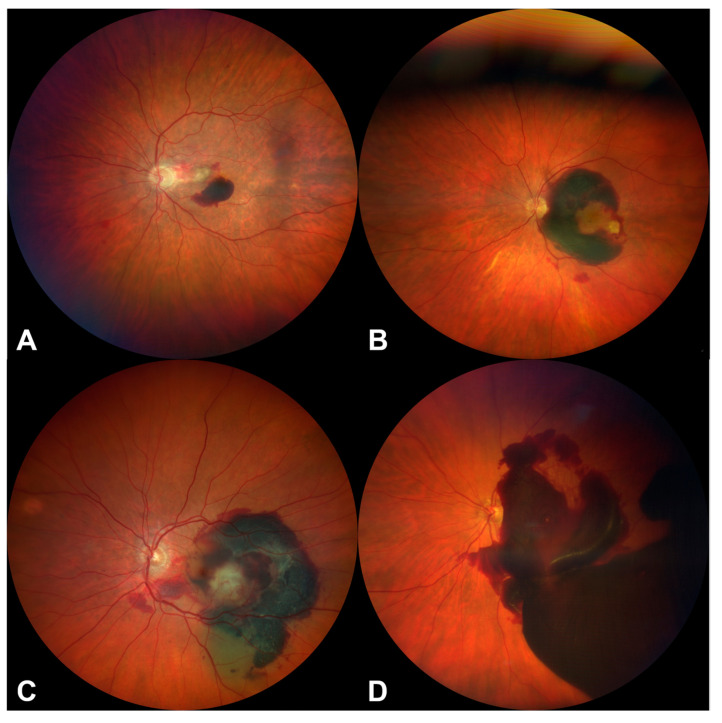
Examples for the classification of the size of macular hemorrhages. (**A**): Small-sized macular hemorrhage (SMH ≤ 4 disc diameters), (**B**): Medium-sized macular hemorrhage (SMH > 4 disc diameters, not extending beyond the vascular arcades), (**C**): Large-sized macular hemorrhage (SMH extends beyond the temporal vascular arcades, but not past the equator), (**D**): Massive sized macular hemorrhage (SHM extends past the equator in at least two quadrants); (**A**,**B**): Group 1, (**C**,**D**): Group 2.

Additionally, we divided the patients into two groups according to the size of SMH. Small- and medium-sized hemorrhages were compiled as “Group 1”, and large and massive hemorrhages as “Group 2” (Figure 1). 

### 2.4. Statistical Analysis

The statistical analysis was supported by the Institute for Biometry and Clinical Research of the University of Muenster. 

Results are presented as absolute and relative frequencies for categorical variables and as median and 25%-and 75%-percentile. A test for cyclic trends in incidence data as well as Chi-Square Test for Specified Proportions were used to compare the occurrence of macular hemorrhages between seasons and months [14]. Fisher’s Exact Test was used to evaluate the influence of season, anticoagulant and antiplatelet medication, and arterial hypertension on the size of the hemorrhage. *p*-values below 0.05 were considered significant. All analyses are explorative and should be interpreted accordingly. Statistical analysis was performed using SAS (Version 9.4, SAS Institute, Cary, NC, USA) and R (Version 4.0.2, R Foundation, Vienna, Austria).

## 3. Results

### 3.1. Subject Characteristics

164 eyes of 164 patients met the inclusion criteria, of which 115 were female and 49 were male. The average age was 81 years. The three most common causes of macular hemorrhage were, in descending order, nAMD (90%), retinal macroaneurysms (3%), and myopic macular neovascularization (3%). A total of 61% of all patients were taking AC/AP medication, whereas 62% of all patients had arterial hypertension as a concomitant disease. The characteristics of the study population are summarized in Table 1. 

Approximately two-thirds of participants were diagnosed with either small or large macular hemorrhages, while medium-sized hemorrhages occurred in approximately one-quarter of patients. Massive hemorrhages occurred in thirteen cases. The number of macular hemorrhages according to size is summarized in Table 2. 

### 3.2. Primary Outcome

**Figure 2:**
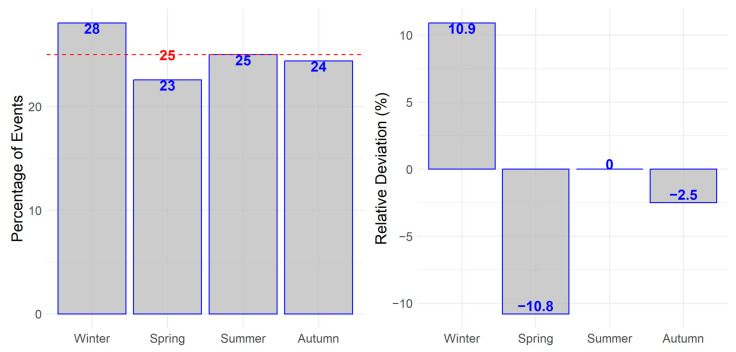
Variations in the incidence of macular hemorrhages in the study population by season. December, January, and February were defined as Winter, March, April, and May as Spring, June, July and August as Summer, September, October and November as Autumn. The red line resembles the expected value under uniform distribution.

Statistical analysis showed no significant seasonal variations in the incidence of SMH in neither “Test for cyclic trends in incidence data” (*p* = 0.81), or in the Chi-Square Test against a uniform distribution of 25% (*p* = 0.80). A relative accumulation of hemorrhages was observed for the winter season, followed by relative troughs during spring and autumn as seen in Figure 2. 

**Figure 3:**
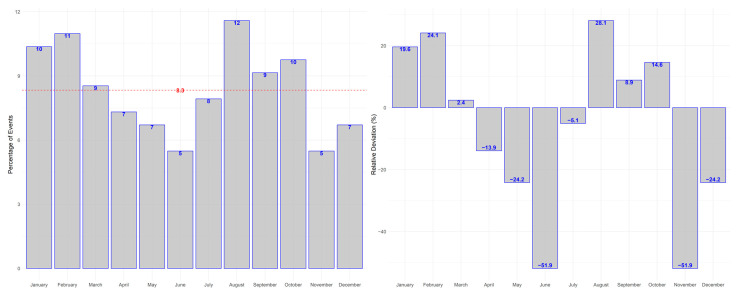
Variations in the incidence of macular hemorrhages in the study population by month. The red line resembles the expected value under uniform distribution.

Seasonal changes were further investigated using a month-based approach. This revealed a wave-like pattern in the monthly distribution of macular hemorrhages, as depicted in Figure 3. However, differences between months were not statistically significant; neither in the “Test for cyclic trends in incidence data” (*p* = 0.82), nor in Chi-Square Test against a unifom distibution of 8.3% (*p* = 0.59).

### 3.3. Secondary Outcome

Group 1 consisted of 101 patients of whom 59 had small and 42 had medium-sized SMH, Group 2 consisted of 63 patients of whom 50 had large and 13 had massive SMH. Small and medium-sized SMH occurred with comparable frequency in the four seasons, whereas most large and massive SMHs occurred in winter (n = 23). The seasons did not affect the size of the hemorrhage with statistical significance (*p* > 0.05). The proportion of hypertensive patients in Group 1 (69%) was slightly smaller than the proportion of hypertensive patients in Group 2 (77%). No significant influence of arterial hypertension on the size of SMH was found (*p* > 0.05). Twenty-three patients were excluded from evaluation regarding this issue as it could not be conclusively determined whether antihypertensive medication was being taken or not.

The proportion of patients on blood-thinning medication was 54% in Group 1 and 71% in Group 2. Intake of AC/AP medication significantly (*p* = 0.03) influenced the size of SMH. The results of the secondary objective are summarized in Table 3. 

## 4. Discussion

This study analyzed seasonal variations in the incidence of macular hemorrhages in a Caucasian population over the course of a six-year time span. Despite a winter-dominant prevalence and a wave-like pattern, acute SMHs did not show statistically significant seasonal variations in a European cohort. As a secondary objective, we found that the intake of AC or AP medication resulted in a significantly larger size of the SMH, whereas arterial hypertension as a concomitant disease and season of occurrence had no statistically significant influence on the size of the hemorrhage.

Seasonal fluctuations have been documented for a variety of different diseases across a broad range of populations and climates. In particular cardiovascular [6,7] and cerebrovascular diseases [8,9] have shown a ’winter peak´ and a through in summer. This has been attributed to changes in ambient temperature, as mentioned morbidities were negatively correlated with it. Fluctuations in blood pressure are also believed to play a role in the development of seasonal variations, as systemic perfusion is subject to temperature-dependent variations with peaks in winter [10]. 

Similarly, a significant winter-dominant prevalence and negative correlation with an ambient temperature of acute SMHs due to AMD was found in Japanese patients with arterial hypertension [11]. Since both SMHs and systemic vascular diseases show the same cyclic trend, the authors hypothesized that similar factors may play a role in the development of such pathologies [11]. 

Conversely, the present study did not show any seasonal variation of SMH in a tertiary eye care center in Germany. However, the comparability of both studies is limited, as there are differences in the prevalence of AMD subtypes, and more precise polypoidal choroidal vasculopathy (PCV) in the studied cohorts [15]. The occurrence of PCV, classified as macular neovascularization (MNV) type 1, is significantly higher in Asian compared to European populations [15,16]. Coscas et al. described PCV, characterized by polypoidal choroidal vascular dilation, to occur more often in Japanese patients (48%) than in French (9%) [15]. The aneurysmal dilations in turn are known to increase the risk of vessel ruptures [17]. Cho et al. found that PCV led to a threefold increase in the incidence rate of massive SMH compared to MNV type 2 nAMD [18]. Interestingly, polypoidal lesions are seen to pulsate [19] suggesting that the structure is influenced by blood pressure. Thus, transient increases in blood pressure, as they are known to occur in the cold [10] might partially disrupt or completely rupture a retinal vessel, causing SMH [20] and thereby contribute to the observed seasonality with peaks in winter [11]. In contrast to that, MNV type 2 in AMD, which is the predominant subtype of European AMD patients [15], is composed of leaky vessels which may make subretinal hemorrhage due to blood pressure fluctuations less likely [18]. Consequently, the higher incidence of PCV in Asia and its risk for SMH and susceptibility to blood pressure fluctuations could explain the seasonal variations found in the Japanese study.

In a more recent trial, which covered a time span similar to the one of this study, the same group observed no seasonal variations [12], which is in line with the results presented here. The authors explain the loss of seasonality by broader access to OCT diagnostics and anti-VEGF drugs [12]. In both Japan and Germany, OCT technology and anti-VEGF treatment were widely available in these years. The authors claim that early detection of MNVs leads to faster treatment initiation, which in turn preventes macular bleeding in the case of AMD. Earlier detection and treatment of PCV minimizes the risk of SMH occurrence due to blood pressure fluctuations and might thus conceal seasonal variations. However, despite the differences in the prevalence of AMD subtypes in both Japan and Germany, the impacts of widespread OCT examinations and anti-VEGF drugs may have been greater than the influences of seasonal variation [12]. 

Consequently, macular hemorrhages appear to be sporadic and not subject to seasonal variations. Another interesting finding is that more extensive SMHs come along with worse visual acuity outcomes [21], therefore interest in the identification of factors that influence the size of the hemorrhage has developed recently. We found that patients with AC and AP medication featured significantly larger SMH, which is in line with previous studies [21,22,23]. In a retrospective analysis of 71 AMD patients, Kuhli-Hattenbach et al. describe that patients receiving antithrombotic therapy had significantly larger hemorrhages than patients who did not receive AC or AP therapy [22]. In a similar approach, Tilanus et al. investigated the influence of AC and AP therapy on hemorrhages in two AMD cohorts. One cohort consisted of patients with small, and the other of patients with massive hemorrhages. They describe that while the intake of antiplatelet agents was not associated with bigger retinal bleedings, the intake of anticoagulant agents (warfarin sodium) resulted in a significant increase in risk for the development of massive hemorrhages [23].

In synopsis, these reports as well as the observations made in this study imply that close cooperation between general practitioners and ophthalmologists is necessary to critically evaluate the indication for AC/AP medication in AMD patients. 

Arterial hypertension on the other hand did not influence the size of SMH, which is in line with the reports by Kuhli-Hattenbach et al. [22]. Yet, the same authors found that arterial hypertension itself is a strong risk factor for large subretinal hemorrhages in AMD patients receiving AC or AP agents [22], which has not been analyzed in the current study. Finally, we did not find a significant association between the season of occurrence and the size of the hemorrhage.

### Limitations

This study is limited by its retrospective nature as well as by its sample size. Both factors can limit its validity regarding future predictions. Prospective multicentric studies with larger cohorts in different countries by different research groups are required for adequate prognoses. 

As was highlighted by the contrary conclusions drawn from both Japanese studies, the time span covered in these retrospective trials has to crucially be considered when interpreting results. Diagnostic and therapeutic technologies of the first decade of the 21st century were different from the ones of the following years. Therefore, the observations made in this trial might also change in association with diagnostic and therapeutic advances in the upcoming years. 

Aside from the vast majority of patients (90%) who suffered from macular hemorrhage due to nAMD, other underlying diseases were included in this study. Though the waiving of the inclusion of only one pathology offers a more realistic approach to the topic at hand, the variety of retinal diseases included in this analysis might have had an impact on seasonality and thus influenced the results. 

## 5. Conclusions

To the best of our knowledge, this is the first trial to analyze the influence of seasonal variations on the incidence of macular hemorrhages in a European cohort. Despite a wave-like pattern in monthly incidence, significant seasonal variations were not identifiable. While the season of occurrence and systemic arterial hypertension as a concomitant disease did not show a significant influence, the intake of AC/AP medication significantly influenced the size of SMH. We, therefore, advise the monitoring of these patients closely, especially in oculus melior situations with nAMD.

## Figures and Tables

**Table 1:** Baseline characteristics of the study population. Values are reported as absolute numbers or as median (25% quartile, 75% quartile).

Parameter	Value
no. of patients	164
median age at onset of hemorrhage (25th and 75th quantile)	81.3 (75.4; 85.9)
gender (f; m)	115; 49
laterality (OD; OS)	81; 83
no. of patients taking AC/AP medication	100
no. of patients with arterial hypertension	102
**cause of macular hemorrhage**	
nAMD	147
macroaneursym	7
myopic CNV	5
secondary CNV due to macular teleangiectasia	2
secondary CNV due to chorioretinits	1
cause unknown	2

no. = number of, f = female, m = male, OD = right eye, OS = left eye, AC/AP medication = anticoagulatory or antiplatelet medication, nAMD = neovascular age-related macular degeneration, CNV = choroidal neovascularization.

**Table 2:** Number of macular hemorrhages according to size. Values are reported as absolute numbers (%).

Group 1		Group 2	
101 (62%)		63 (38%)	
Small	Medium	Large	Massive
59 (36%)	42 (26%)	50 (30%)	13 (8%)

**Table 3:** Influence of seasons, arterial hypertension, and anticoagulatory/antiplatelet medication on the size of macular hemorrhages. *p* values < 0.05 are highlighted in bold.

	Group 1 n (%)	Group 2 n (%)	*p* Values
seasons			0.19 *
winter	23 (14.02%)	23 (14.02%)	
spring	27 (16.46%)	10 (6.10%)	
summer	25 (15.24%)	16 (9.76%)	
autumn	26 (15.85%)	14 (8.54%)	
arterial hypertension			0.34 *
yes	58 (41.13%)	44 (31.21%)	
no	26 (18.44%)	13 (9.22%)	
AC/AP medication			0.03 *
yes	55 (33.54%)	45 (27.44%)	
no	46 (28.05%)	18 (10.98%)	

Group 1 = small/medium hemorrhages; Group 2 = large/massive hemorrhages. AC/AP medication = anticoagulatory or antiplatelet medication, n = number, % = percentage. * Fisher’s Exact Test.

## Data Availability

Not applicable.
